# Morphological Analysis and Short-Term Evolution in Pulmonary Infarction Ultrasound Imaging: A Pilot Study

**DOI:** 10.3390/diagnostics16030383

**Published:** 2026-01-24

**Authors:** Chiara Cappiello, Elisabetta Casto, Alessandro Celi, Camilla Tinelli, Francesco Pistelli, Laura Carrozzi, Roberta Pancani

**Affiliations:** 1 Pulmonary Unit, Cardiothoracic and Vascular Department, Pisa University Hospital, 56124 Pisa, Italy; chiara.cappiello92@gmail.com (C.C.); milla.tinelli@gmail.com (C.T.); francesco.pistelli@unipi.it (F.P.);; 2 Department of Surgical, Medical and Molecular Pathology and Critical Care Medicine, University of Pisa, 56124 Pisa, Italy

**Keywords:** pulmonary infarction, pulmonary embolism, lung ultrasound, time evolution

## Abstract

**Background:** Pulmonary infarction (PI) is the result of the occlusion of distal pulmonary arteries resulting in damage to downstream lung areas that become ischemic, hemorrhagic, or necrotic, and it is often a complication of an underlying condition such as pulmonary embolism (PE). Since in most of cases it is located peripherally, lung ultrasound (LUS) can be a good evaluation tool. The typical radiological features of PI are well-known; however, there are limited data on its sonographic characteristics and its evolution. **Methods:** The aim of this study is to evaluate, using LUS, a convenience sample of patients with acute PE with computed tomography (CT) consolidation findings consistent with PI. Patients’ clinical characteristics were collected and LUS findings at baseline and their short-term progression was assessed. LUS was performed within 72 h of PE diagnosis (T0) and repeated after one (T1) and four weeks (T2). Each procedure started with a focused examination of the areas of lesions based on CT findings, followed by an exploration of the other posterior and lateral lung fields. The convex probe was used for initial evaluation integrating LUS evaluation with the linear one was employed for smaller and more superficial lesions and when appropriate. Color Doppler mode was added to study vascularization. **Results:** From June to October 2023, 14 consecutive patients were enrolled at the Respiratory Unit of the University Hospital of Pisa. The main population characteristics included the absence of respiratory failure and prognostic high-risk PE (100%), the absence of significant comorbidities (79%), and the presence of typical symptoms, such as chest pain (57%) and dyspnea (50%). The average number of consolidations per patient was 1.4 ± 0.6. Follow-up LUS showed the disappearance of some consolidations and some morphological changes in the remaining lesions: the presence of hypoechoic consolidation with a central hyperechoic area (“bubbly consolidation”) was more typical at T1 while the presence of a small pleural effusion often persisted both at T1 and T2. A decrease in wedge/triangular-shaped consolidations was observed (82% at T0, 67% at T1, 24% at T2), as was an increase in elongated shapes, representing a residual pleural thickening over time (9% at T0, 13% at T1, 44% at T2). A reduction in size was also observed by comparing the mean diameter, long axis, and short axis measurements of each consolidation at the three different studied time points: the average of the short axes and the median of the mean diameters showed a statistically significant reduction after four weeks. Additionally, a correlation between lesion size and pleuritic pain was described, although it did not achieve statistical significance. **Conclusions:** Patients’ clinical characteristics and ultrasound features are consistent with previous studies studying PI at PE diagnosis. Most consolidations detected by LUS change over time regarding size and form, but a minority of them do not differ. LUS is a safe and non-invasive exam that could help to improve patients’ clinical approach in emergency rooms as well as medical and pulmonology settings, clinically contextualized for cases of chest pain and dyspnea. Future studies could expand the morphological study of PI.

## 1. Introduction

Pulmonary infarction (PI) is a consequence of the occlusion of distal pulmonary arteries, resulting in damage to downstream lung areas that become ischemic, hemorrhagic, or necrotic [1]. The lung’s dual arterial supply, provided by both the bronchial arteries and pulmonary circulation, exhibits protective action against the threat of ischemia [2,3]. However, this protective mechanism may fail, and conditions for PI development may occur [4]. In most cases, PI represents a complication from an underlying condition, such as a pulmonary embolism (PE), infection, or neoplasia; its incidence has a very wide range, possibly due, at least in part, to its different radiological, clinical, and anatomopathological definitions [5]. PI is often associated with pleural effusion, which, from a pathophysiological point of view, probably forms due to an increased permeability of the pulmonary capillaries through the release of inflammatory mediators, such as vascular endothelial growth factor, from platelet-rich thrombi. Despite the fact that the literature on PI is limited, and no specific guidelines are available [1], its epidemiological and clinical relevance is not negligible, given that autopsy studies have identified PI in more than 50% of PE deaths [6].

The typical radiological features of PI are well-known and are described as the so-called Hampton’s hump, a wedge-shaped consolidation at the periphery of the lung, visible in a standard chest X-ray and even more precisely with a computed tomography pulmonary angiography (CTPA) [7,8,9]. In the latter case, the diagnosis is based on the presence of a peripheral wedge-shaped consolidation within the region of an obstructed vessel, either associated or not with other suggestive findings like vessel signs, central lucency, and absence of an air bronchogram. Because PIs are typically in contact with the pleura, a lung ultrasound (LUS) has the potential to be a useful tool for their evaluation at diagnosis [4,10,11,12]. Due to its safety and widespread availability, it is also conceivable that LUS might be useful for monitoring the evolution of a PI during hospitalization and over a long-term clinical course. However, in contrast to the relative abundance of information available regarding the radiological features of PI, LUS imaging has been explored to a much lesser extent, although interest has increased over the past few years [13]. The typical LUS finding for PI is a wedge-shaped, hypoechoic, pleural-based parenchymal consolidation, extending to the pleural surface, and localized where the patient feels pleuritic pain. Less frequently, PI may be round/ovoid or polygonal in shape [4]. Copetti et al. recently described a “surviving lung” as a highly specific LUS sign of PI: a triangular hypoechoic consolidation, in the absence of air bronchograms, and a mostly central, roundish hyperechoic area. The authors hypothesized that a lucency within a PI consolidation may indicate the coexistence of aerated non-infarcted and infarcted lung tissues in the same lobule [14].

The aim of this study was to further characterize the LUS aspects of PI and to investigate the potential role of this technique in monitoring the condition over time.

## 2. Materials and Methods

**Figure 1:**
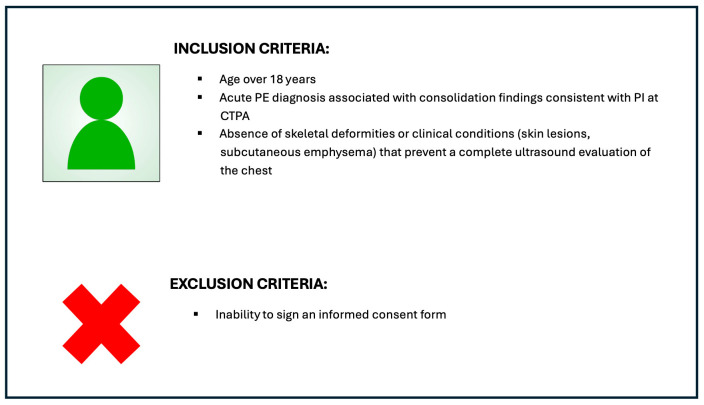
Inclusion and exclusion criteria.

This was an observational, single-center, pilot study. Inclusion and exclusion criteria are described in Figure 1. Authorization from the ethics committee was not required because the study did not change the standard clinical management of patients. Patients were asked to provide generic informed consent for clinical management and for the collection of anonymized clinical data. Since all the procedures described in this study were part of the routine care of patients, this study was conducted in compliance with the Declaration of Helsinki without the need for local ethics committee approval. A convenience sample of patients with acute PE associated with CTPA consolidation findings consistent with PI discovered during their clinical course at the hospital and/or short-term follow-up (F-U) was enrolled. Our CTPA protocol called for bolus tracking with a region of interest on the common trunk of the pulmonary artery, with a flow rate of 3–5 milliliters per second of the contrast agent, a one-millimeter-thick transverse CTPA section, and 80–140 kV tube voltage according to body mass index, using the shortest rotation time and the thinnest collimation to obtain high quality images [15].

Smoking habits, symptoms, comorbidities, anticoagulant therapy, and PE prognostic markers (troponin and right ventricular dysfunction signs detected by CTPA) were collected from all patients. The characteristics of the consolidations and their short-term changes were evaluated by a pulmonologist with 5 years of expertise in LUS, with the procedure being as follows: patients underwent baseline LUS evaluation within 72 h of PE diagnosis (T0), repeating it at one (T1) and four weeks (T2). T0 and T1 were performed in an inpatient setting, while T2 was performed during an outpatient visit. Each patient was always examined by the same operator to eliminate inter-observer bias. A GE Medical System LOGIQ v2 or GE Medical System LOGIC ultrasound scanner (both from GE Medical, Little Chalfont, Buckinghamshire, UK) was used for all the procedures. Each instrument was preset in terms of probe, depth, gain, and time gain compensation, and the probe landmark was aligned on the same side as the monitor marker to obtain conventionally comparable images. The evaluation was performed with the patient sitting and breathing spontaneously; during image acquisition, a breath-hold of a few seconds was required to minimize artifacts from breathing-related movements. The LUS images were obtained using a convex probe (3.5–5 megahertz) placed in a transverse approach, using a depth of approximately 8 cm and focusing on the pleural line, and a linear probe (7.5–10 megahertz), with a depth of approximately 3 cm. Each procedure began with a focused PI examination, assessing the anatomic area of the lesion at CTPA, followed by a panoramic exploration of the other posterior and lateral lung fields. As LUS was performed in an urgent setting, we excluded the anterior chest fields to reduce the examination time and patient discomfort, as suggested by current guidelines [16]. A convex probe was used for the initial evaluation; a linear probe was then used, deepening the visualization of smaller lesions when appropriate. For each lesion, color Doppler mode was applied with the purpose of studying the vascularity of the identified consolidations, and the patient was asked to maintain a few seconds of apnea to minimize motion artifacts resulting from respiration. Data were recorded on the shape (wedge/triangular, polygonal, roundish), size (long axis, short axis, and mean diameter, the latter calculated as the sum of the first two measurements divided by two), and echogenicity (hypoechogenic, isoechogenic, hyperechogenic), as well as the presence of air bronchograms or hyperechogenic punctiform spots within the lesion (“bubbly consolidation”). Of note, the term “bubbly consolidation” was coined by Copetti et al. [14], but it is not widely mentioned in the current literature; for example, the most recent guidelines on LUS [16] do not use this term. In addition, the presence of pleural effusions was reported, and tissue vascularization was studied by color Doppler modality.

### Statistical Methods

Data distribution was analyzed using the Shapiro–Wilk test. Values with a normal distribution are presented as mean ± standard deviation (SD). For values not normally distributed, the median and interquartile range (IQR) are shown. Differences between means were calculated using ANOVA or Friedman’s test as appropriate. Student’s *t*-test was used to compare the means of two independent samples. Values of *p* < 0.05 were considered statistically significant. Graphs were obtained with Prism^®^ software (Version 10 for MacOS; GraphPad, San Diego, CA, USA).

## 3. Results

**Figure 2:**
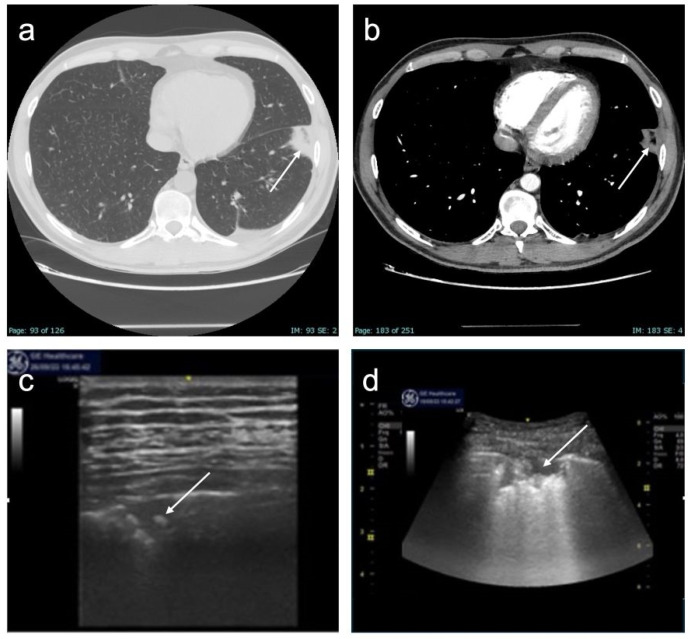
Example of PI imaging by LUS: panel (**a**,**b**,**d**): a subpleural consolidation at T0 with CTPA and LUS; panel (**c**) a subpleural consolidation characterized by the presence of an air bronchogram or hyperechogenic punctiform spot within the lesion (“bubbly consolidation”). The arrow indicates the image of PI by CTPA in panels (**a**,**b**), by LUS in panel (**d**), and “bubbly consolidation” interno al PI in panel (**c**).

**Figure 3:**
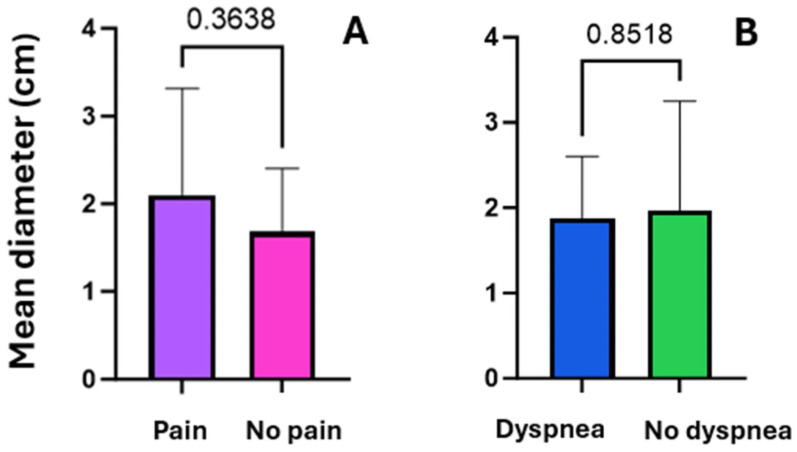
Comparison of the subpopulations (**A**): pleuritic pain vs. non-pleuritic pain; (**B**) dyspnea vs. non-dyspnea) regarding the mean diameter of PIs.

**Figure 4:**
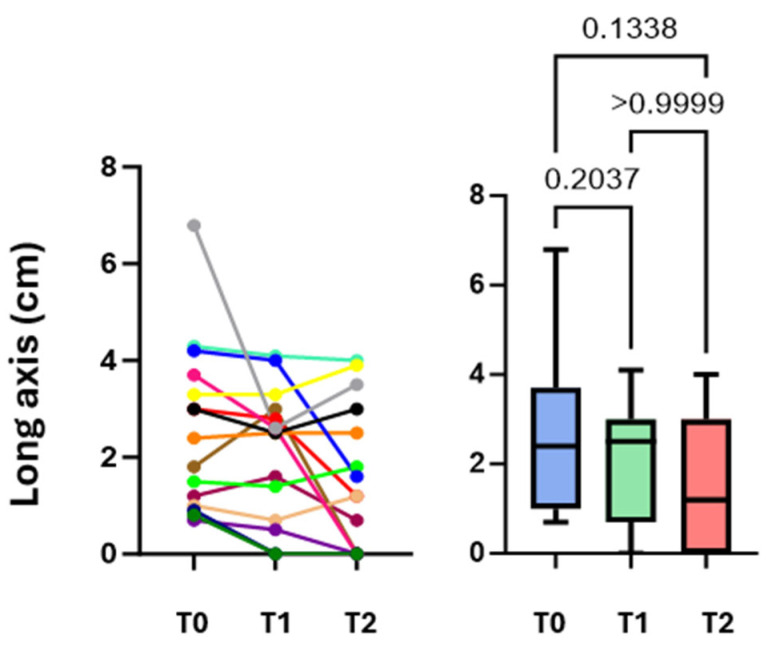
Comparison of long axis measurements at T0, T1, and T2. The colored lines correspond to the individual patients evaluated at T0-T1-T2.

**Figure 5:**
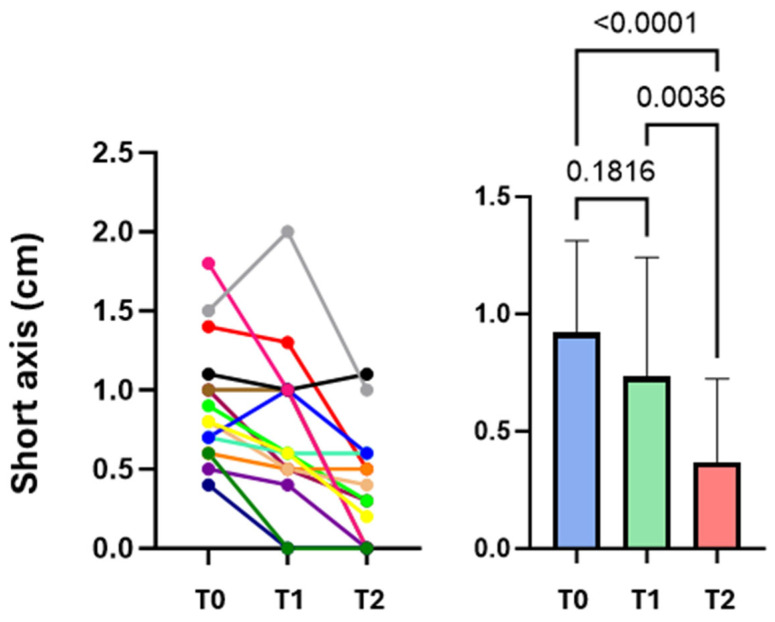
Comparison of short axis measurements at T0, T1, and T2. The colored lines correspond to the individual patients evaluated at T0-T1-T2.

**Figure 6:**
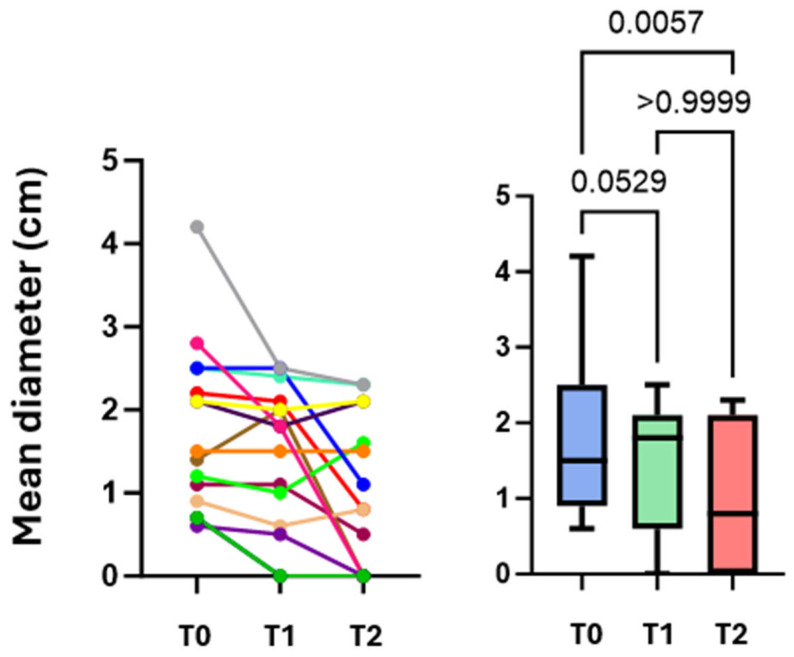
Comparison of mean diameters measured at T0, T1, and T2. The colored lines correspond to the individual patients evaluated at T0-T1-T2.

Fourteen patients admitted to the Respiratory Unit of Pisa University Hospital from June to October 2023 were enrolled. The characteristics of the population are shown in Table 1. The most frequently reported symptoms were chest pain (eight cases, including seven cases of localized pleuritic pain and one case of atypical chest pain), dyspnea (seven cases), hemoptysis (three cases), and syncope/presyncope (two cases). No patients were in respiratory failure. None of the patients had hemodynamic instability, so none were classified as high risk for 30-day mortality according to the 2019 ESC guidelines for pulmonary embolism, although 12 (86%) and 2 (14%) patients were classified as low-intermediate and high-intermediate, respectively [17]. CT scans showed no PI in the anterior fields. Of the 14 patients examined at T0, 10 performed full F-Us at T1 and T2; 2 patients performed only a T1 F-U and were not recalled to the outpatient setting due to poor clinical conditions that would have made a visit inconvenient; and 2 patients underwent only a T2 F-U because they had already been discharged less than 7 days after baseline. The mean number of subpleural consolidations per patient was 1.4 ± 0.6; 22 consolidations were found at T0, 15 at T1, and 16 at T2. These results are due to the resolution of seven consolidations from T0 to T1 and the appearance of only one new consolidation from T1 to T2. Lesions were mainly located in the posterior basal lung fields (59%) and left hemithorax (59%). Almost all subpleural consolidations (91%) had a hypoechogenic pattern. All PIs diagnosed with CT scans were detected by LUS at T0 (100% sensitivity). During F-U, there was an evolution in the morphology of some lesions, sometimes with the persistence of only elongated pleural thickenings (44%). Most lesions had a “sentinel”, limiting pleural effusion (68% at T0, 80% at T1, and 69% at T2). Some lesions appeared as bubbly consolidations, mostly at T1 compared with T0 (60% vs. 27%); at T2, only 12% of lesions still appeared as bubbly consolidations (Figure 2). The most prevalent PI form on LUS at T0 was wedge or triangular (82%). A vascular sign was described at the apex of three consolidations at T0 (14%), and at the apex of only one consolidation at T1 (7%) and T2 (6%). As for the vascular sign, a comparison of CTPA images with ultrasound images at T0 was made, and an equivalent sensitivity was found (14% in both cases). Consolidation measurements (short axis, long axis, mean diameter) are shown in Table 2. The values of mean diameter and long axis did not show a normal distribution, unlike those of the short axis. To assess whether the size of the consolidations conditioned the presence of pleuritic pain, the mean diameters of the consolidations were compared between patients who presented with pleuritic pain and those who did not. There was no statistically significant difference between the two groups (*p* = 0.364; Student’s *t* test), even though a trend toward a larger size of the consolidations was shown in patients who presented with pleuritic pain (Figure 3A). Similarly, the mean diameter at baseline was compared with the presence or absence of dyspnea. Again, there was no statistically significant difference between the two subpopulations examined (*p* = 0.852; Student’s *t* test) (Figure 3B). Next, the trend in the size of consolidations during F-U was analyzed. Most of them decreased in size, and others disappeared (25% of the thickenings disappeared at T2). Only measurements obtained in patients who underwent all three ultrasound checks were compared. Using Friedman’s test, the difference between the long axes’ medians was not statistically significant. However, a downward trend emerged at T2 (Figure 4). The ANOVA test did not show a significant difference between the short axes’ means at T0 and T1 (*p* = 0.2037); in contrast, the difference was statistically significant when comparing the mean at T1 with the mean at T2 (*p* = 0.0036) and, more importantly, when comparing the mean at T0 with the mean at T2 (*p* < 0.0001) (Figure 5). Finally, using Friedman’s nonparametric test, a significant reduction in mean diameter was demonstrated between T0 and T2 (*p* = 0.0057) (Figure 6).

## 4. Discussion

CTPA is the most commonly used imaging technique to diagnose PI, and radiological findings suggestive of PI include a feeding vessel or “vessel sign,” a central hyperlucency, and a semicircular shape [18]. The former represents the presence of an enlarged vessel at the apex of the consolidation [13,19]. Central lucency within a peripheral consolidation is also particularly suggestive of PI; however, other conditions can also present this feature, such as cavitation, cysts, or dilated airways [2]. Another suggestive finding is the absence of an air bronchogram; if present, it almost certainly points to a different diagnosis. Therefore, the presence of a vessel sign on CT images and of an area of central brightness associated with the absence of an air bronchogram has a very high specificity (99%) [18]. LUS has played a pivotal role in clinical practice for several years now, representing a low-risk, rapid, and easy-to-perform tool for the evaluation of the respiratory system of a patient. In suspected PE, the performance of CTPA is imperative (unless specifically contraindicated); however, this examination is not easily repeated in short-term F-U. CTPA remains the gold standard according to the current European guidelines for the diagnosis of PE and pulmonary vasculature evaluation [17]. It is readily available and rapid, providing a panoramic view of the chest that is not possible with LUS; however, the radiological impact of multiple exams over time is significant.

While LUS can be a complementary tool during follow-up, especially in intensive care unit patients, it has limitations due to variations in physician expertise, skeletal deformities, and clinical conditions that strongly limit its utility, such as subcutaneous emphysema and the lack of a panoramic view. However, it has been demonstrated that the learning curve for this method is short in patients with acute dyspnea [20]. Patients with PE may present with entirely negative ultrasonographic evaluation, but if PE is associated with PIs, these may be visualized by LUS. The ultrasonographic features of PIs are known, and knowing how to recognize them can be useful in the presence of subpleural consolidation [10,11,16]. In this study, we recruited 14 patients who had subpleural thickening or consolidation compatible with PIs, comparing the characteristics of the population and their respective lesions with the data in the literature and following them over time. The population examined had a mean age of slightly above 50 years; they were mostly healthy patients before the thromboembolic event, and in almost all cases, the event was at low or intermediate-low mortality risk. The characteristics of the population agree with previous studies performed by Kaptein, Miniati, and Islam, which demonstrated a higher frequency of PI in young patients without comorbidities [1,19,21]. Most of the patients examined had typical symptoms such as pleuritic pain and dyspnea. It appears in the literature that in the presence of such symptoms, recovery from the disease is slower. We wondered whether patients with pleuritic pain and dyspnea had PIs with larger dimensions, but no statistically significant difference was found compared with patients without these symptoms. However, there seems to be a tendency for patients with pain to have PIs with a larger size, so a larger population would be needed to rule out that this correlation is related to chance. Consistent with what is found in the literature, shape and size tend to vary over time. At T0, a wedge-shaped/triangular morphology predominates, which is typical, in fact, of newer PIs.

Subsequently, we found that the percentage of thickenings with polygonal shapes, typical of older PIs, increased, but above all, elongated shapes, characteristic of subpleural thickening or the expression of a residual fibrotic scar, increased. Changes in morphology were inevitably followed by changes in measurements; in fact, the mean diameter and long axis showed a reversal of the trend, with an increase in their medians at T1. The greatest change, however, emerged at T2, when most thickening was significantly reduced from baseline in almost all dimensions (in a statistically significant manner for mean diameter and short axis). These results suggest that ultrasound at baseline could be performed in clinical practice to be compared directly with ultrasound after one month, omitting the intermediate F-U. However, ultrasound at one week showed the disappearance of some thickening and sometimes morphologic changes; for example, central hyperlucency was more frequent at one week; in fact, sometimes this finding correlates with older PIs [11]. The association with a limited pleural effusion in our patients often persisted after four weeks. In fact, based on some studies, the pleural effusion associated with PE disappears later in the presence of PI than in PE without PI (on average 2–3 weeks) [22]. Finally, in some cases, a vascular sign is present, which, when compared on ultrasound images and CT scans, does not seem to point toward different sensitivities between the two examinations in recognizing this finding. This study has several limitations: the first one is the small sample size, which is further reduced if we consider the few cases that underwent all three ultrasound examinations. The second limitation is the performance of an operator-dependent examination in a single center, by a single examiner, and sometimes with two different instruments. The third limitation is related to the choice to exclude the anterior chest fields from the examination, which was made to reduce the examination time and patient discomfort; however, the probability of having missed anteriorly located PIs is minimal when considering that CT scans were evaluated before performing the examination, and no anterior PIs were observed. The last limitation is related to the risk of not having sufficiently detected pulmonary thickening hidden behind the bones; this limitation, however, is intrinsic to the ultrasound method itself. Despite these limitations, our work might lead to a better understanding of the evolution of PI without the need for follow-up chest X-rays and/or CT scans, with a significant reduction in costs and in diverse settings, including outpatient settings.

## 5. Conclusions

So far, very few data are available on the evolution of PIs; from the literature, it appears that they decrease over time and, in some cases, disappear. In our study, most of the lung thickenings or consolidations experienced this evolution, but a minority of cases retained the same shape, size, or collateral findings. In clinical practice, we do not actually know what the evolution of each of these findings is, and it is not known what the ideal timing for ultrasound F-U might be, with a focus on ruling out the possibility that lesions of a different nature are hidden behind some of these thickenings. Knowing how to use a tool such as LUS in this area could add value to the management of this pathology. While limited by the small sample size, the study has the potential to provide a better understanding of PI evolution with LUS, which has the potential to reduce the need for repeated CT scans. Further studies will be needed to investigate these aspects further.

## Figures and Tables

**Table 1:** Patients’ characteristics.

Male Sex (%)	85
Age, years (mean ± SD)	56 ± 15
BMI, kg/m^2^ (mean ± SD; missing)	30 ± 9; 3
Smoking habit (%)	
- Current smokers	15
- Former smokers	70
- Never smokers	15
Cardiovascular comorbidities (%)	21
Respiratory comorbidities (%)	21
Chest pain (%)	57
Pleuritic (%)	87
Atypical (%)	13
Dyspnea (%)	50
Hemoptysis (%)	21
Syncope (%)	14
Hemodynamic instability (%)	0
CTPA Right ventricular dysfunction (%)	86
Dimer D, ng/mL FEU (median [IQR])	10,870 [2293–16,435]
Troponin HS, ng/L (median [IQR]) *	7.5 [5–43.7]
C-reactive protein (C-RP), mg/dL (median [IQR])	4.7 [2.3–9.6]
Respiratory failure (%)	0
First anticoagulant therapy (%)	
- Low molecular weight heparin/fondaparinux	93
- Unfractionated heparin	7
Anticoagulant therapy at discharge (%)	
- Direct oral anticoagulants	86
- Low molecular weight heparin	7
- Warfarin	7

* Normal values: Troponin HS ≤ 14 ng/L, Dimer D ≤ 500 ng/mL FEU, C-RP ≤ 0.50 mg/dL.

**Table 2:** Ultrasonographic characteristics of consolidations.

	T0 (N = 22)	T1 (N = 15)	T2 (N = 16)
Morphology			
Wedge/triangular, N (%)	18 (82)	10 (67)	4 (25)
Polygonal, N (%)	0	1 (7)	4 (25)
Round, N (%)	2 (9)	2 (13)	1 (6)
Elongated, N (%)	2 (9)	2 (13)	7 (44)
Echogenicity			
Hypoechogenic pattern, N (%)	19 (91)	15 (100)	16 (100)
Hyperechogenic pattern, N (%)	3 (9)	0	0
Margins			
Regular, N (%)	17 (77)	11 (73)	11 (69)
Irregular, N (%)	5 (23)	4 (27)	5 (31)
Sentinel pleural effusion, N (%)	15 (68)	12 (80)	11 (69)
“Bubbly consolidation”, N (%)	6 (27)	9 (60)	2 (12)
Air bronchogram, N (%)	0	1 (7)	0
“Vessel sign”, N (%)	3 (14)	1 (7)	1 (6)
Dimension			
Short axis, cm (mean + DS)	0.9 + 0.4	0.7 + 0.5	0.4 + 0.4
Long axis, cm (median, [IQR])	2.4 [1.0–3.7]	2.5 [0.7–3.0]	1.2 [0–3.0]
Mean diameter, cm (median, [IQR])	1.5 [0.9–2.5]	1.8 [0.6–2.1]	0.8 [0–2.1]

## Data Availability

The raw data supporting the conclusions of this article will be made available by the authors on request.

## Abbreviations

The following abbreviations are used in this manuscript:

PI	Pulmonary infarction
PE	Pulmonary embolism
CTPA	Computed tomography pulmonary angiography
LUS	Lung ultrasound
F-U	Follow-up
CT	Computed tomography
T0	At PE diagnosis
T1	1 week from T0
T2	4 weeks from T0
